# Level of evidence of clinical neurosurgery research in Saudi Arabia

**Published:** 2014-10

**Authors:** Bakur A. Jamjoom, Aimun A. Jamjoom, Abdulhakim B. Jamjoom

**Affiliations:** *From the Department of Trauma & Orthopedics (Jamjoom BA), University Hospital of Coventry and Warwickshire, Coventry, the Department of Clinical Neurosciences (Jamjoom AA), Western General Hospital, Edinburgh, Scotland, United Kingdom, and the Section of Neurosurgery (Jamjoom AB), Department of Surgery, King Khalid National Guard Hospital, Jeddah, Kingdom of Saudi Arabia*

Good quality clinical research is fundamental for the practice of evidence-based medicine (EBM). Level of evidence (LOE) is a tool used to assess the quality and design of clinical research. It is generally accepted that publications with a high LOE are likely to be more valid and have greater impact on clinical practice. In recent years, there has been a growing interest in the assessment of the LOE in publications from the Kingdom of Saudi Arabia (KSA),^1^,^2^ in major specialty journals,^3^,^4^ and from different countries.^5^ Up to date information on the quality of neurosurgery research in KSA is still lacking. This study aimed at evaluating the LOE in Saudi neurosurgical publications, and comparing this with the international literature, and with KSA publications from other specialties.

This study was carried out at the King Khalid National Guard Hospital (KKNGH), Jeddah, KSA between November and December 2013. It was a review based on routinely available open access data; hence, did not require ethical approval by KKNGH. The authors compiled a list of 73 consultant neurosurgeons that worked in KSA over the last 24 years by means of records from the Saudi Association of Neurological Surgery (SANS) membership lists and previous meetings programs. Using the name of each neurosurgeon, “PubMed,” and “Google Scholar” searches were carried out to identify all KSA articles published between 1990-2013. The inclusion criteria were clinical neurosurgery and neurosciences articles published in English in any journal, where the name of at least one KSA neurosurgical center was represented in the authorship. Articles that reported basic research, laboratory work, and letters to editors were excluded.

Using the full publication, the first 2 authors assessed every article independently and discrepancies were resolved by discussion. Every study was ranked according to its LOE using Oxford’s LOE Scale.^6^ The LOE in the articles was evaluated and compared with respect to: the year of publication, whether during 1990-2000 or 2001-2013, the presence or absence of international collaboration, the journal’s category, whether neuroscience (including neurosurgery) or other medical, the journal’s impact factor (IF) whether equal and higher or lower than one, the research topic, whether related to neurosurgery or to other neurosciences and the article’s citation numbers whether equal and higher or lower than 10. The findings were also compared with the LOE in Saudi orthopedics and plastic surgery publications,^1^,^2^ as well as the LOE in spine^3^ and neurosurgery^4^ publications in major international journals, and in articles published by Iranian neurosurgeons.^5^ GraphPad QuickCalcs (GraphPad Software, La Jolla, CA, USA) was used for the statistical analysis. A kappa value was calculated to estimate the level of agreement between the reviewers. The various pairs of LOE findings were examined statistically by comparing the differences in their LOE means and 95% confidence intervals (CI) using unpaired t test. A *p*<0.05 was considered statistically significant.

A total of 377 Saudi clinical neurosurgery articles published during 1990-2013 were identified as suitable for the study. The number of publications per year ranged from 4-27 (mean 16). The LOE of the articles was as follows: I: 1 (0.3%), II: 2 (0.5%), III: 31 (8.2%), and IV: 343 (91%). The level of agreement between the 2 reviewers was good (kappa=0.771). The primary research centers and number of publications were: King Khalid University Hospital (KKUH), Riyadh: 143 (37.9%), King Faisal Specialist Hospital and Research Center (KFSHRC), Riyadh: 87 (23.1%), King Fahad University Hospital, Alkhobar: 47 (12.5%), Prince Sultan Military Medical City, Riyadh: 23 (6.1%), King Abdulaziz University Hospital, Jeddah: 20 (5.3%), King Fahad National Guard Hospital, Riyadh: 17 (4.5%), KKNGH, Jeddah: 14 (3.7%), King Faisal Specialist Hospital and Research Center, Jeddah: 10 (2.7%), King Fahad Medical City, Riyadh: 9 (2.4%), and others 7 (1.9%). There was collaboration with centers in other countries in 41 (10.9%) articles. These countries and the number of articles were: United Kingdom 29, Canada 6, France 3, United States of America one, Japan one, and Australia one. There was also collaboration with other KSA centers in 16 (4.2%) articles. The articles were published in journals with IF ranging from 17.2 to 0.119 (median 1.18). Thirty-four articles were published in journals with an unrecorded IF. The journal’s category and number of articles were: neurosurgical 133 (35.3%), other neurosciences 114 (30.2%), and other medical 130 (34.5%). The most frequently used journals and the numbers of articles were: Neurosciences (Riyadh): 45 (12%), British Journal of Neurosurgery: 35 (9%), Annals of Saudi Medicine: 27 (7%), Childs Nervous System: 25 (7%), Acta Neurochirurgica: 23 (6%), Saudi Medical Journal: 23 (6%), Surgical Neurology: 22 (6%), Neurosurgical Review: 18 (5%), Minimally Invasive Neurosurgery: 8 (2%), Journal of Neurosurgery: 8 (2%), Pediatric Neurosurgery: 6 (2%), Middle Eastern Journal of Anesthesia: 5 (1%), Journal of Pakistani Medical Association: 5 (1%), Neurosurgery: 4 (1%), Canadian Journal of Neuroscience: 4 (1%), and International Journal of Radiation Oncology Biology: 4 (1%). The remaining 115 (31%) articles were published in another 83 journals.

The research types of the various articles were: case reports: 179 (47.5%), retrospective studies and case series: 142 (37.7%), prospective studies including one randomized control trial: 24 (6.4%), epidemiological studies and technical notes: 21 (5.6%), and review articles including 2 systematic reviews: 11 (2.9%). The research topics were related to neurosurgery in 316 (83.8%), and to other neurosciences in 61 (16.2%) articles. The neurosurgery topics were: tumor: 83 (22%), pediatric neurosurgery: 70 (18.6%), spine: 42 (11.1%), infection: 38 (10.1%), vascular: 33 (8.8%), trauma: 29 (7.7%), functional: 12 (3.3%), neurosurgery practice and history: 7 (1.9%), and peripheral nerve: 2 (0.5%). The other neurosciences topics were: neurology: 19 (5%), neuropathology: 9 (2.4%), neuroanesthesia and intensive care: 9 (2.4%), oncology and radiotherapy: 9 (2.4%), neuroradiology: 7 (1.9%), pediatric neurology: 5 (1.3%), and neuropsychology and rehabilitation: 3 (0.8%). The citation numbers of the articles ranged from 0-148 (median 11). One hundred and five (27.8%) articles had no recorded citation number. **Table 1** summarizes the LOE of clinical neurosurgery research in KSA in relation to a number of features including a comparison with the LOE in publications from other Saudi specialties and in the international neurosurgical and spine literature.

**Table 1:** Level of evidence of clinical neurosurgery research in the Kingdom of Saudi Arabia.

Feature	Articles numbers	Level of evidence (LOE), n (%)				LOE mean	Mean difference (95% confidence interval)	*P*-value (significance)
		I	II	III	IV			
***Year***							
1990-2000	204	0	0	12 (5.9)	192 (94.1)	3.94	0.0900 (0.0208 – 0.1592)	0.0110 (Sig)
2001-2013	173	1 (0.6)	2 (1.2)	19 (11.0)	151 (78.3)	3.85		
***International Collaboration***							
Yes	41	0	2 (4.9)	5 (12.2)	34 (82.9)	3.78	-0.1300 (-0.2402 – -0.0198)	0.0209 (Sig)
No	336	1 (0.3)	0	26 (7.7)	309 (92.0)	3.91	
***Journal***							
Neuroscience*	247	1 (0.4)	2 (0.8)	16 (6.5)	228 (92.3)	3.91	0.0300 (-0.0424 – 0.1024)	0.4160 (NS)
Other medical	130	0	0	15 (11.5)	115 (88.5)	3.88	
***Journal’s IF***							
IF ≥1	243	1 (0.4)	1 (0.4)	20 (8.2)	221 (90.9)	3.90	0.000 (-0.0719 – 0.0719)	1.000 (NS)
IF <1	134	0	1 (0.7)	11 (8.2)	122 (91.0)	3.90	
***Research topic***							
Neurosurgery	316	1 (0.3)	2 (0.6)	19 (6.0)	294 (93.0)	3.92	0.1200 (0.0259 – 0.2141)	0.0126 (Sig)
Other neuroscience†	61	0	0	12 (19.7)	49 (80.0)	3.80	
***Article citation numbers***							
Citations ≥10	140	0	2 (1.4)	15 (10.7%)	123 (87.9)	3.86	-0.0600 (-0.1320 – 0.0120)	0.1021 (NS)
Citations <10	237	1 (0.4)	0	16 (6.8%)	220 (92.8)	3.92	
***Comparison with literature***							
Current Study	377	1 (0.3)	2 (0.5)	31 (8.2)	343 (91.0)	3.90		
Makhdom et al^1^ 2013 (Ortho-KSA)	159	4 (2.5)	9 (5.7)	8 (5.0)	138 (86.8)	3.76	0.1400 (0.0540 – 0.2260)	0.0015 (Sig)
Samargandi et al^2^ 2013 (Plastic-KSA)	246	0	6 (2.4)	14 (5.7)	226 (91.9)	3.89	0.0100 (-0.0474 – 0.0674)	0.7322 (NS)
Amiri et al^3^ 2013 (Spine-USA/Europe)	703	33 (4.7)	163 (23.2)	88 (12.5)	419 (59.6)	3.27	0.6300 (0.5286 – 0.7314)	0.0001 (Sig)
Rothoerl et al^4^ 2003 (Neuro-USA/Europe)	441	28 (6.3)	138 (31.3)	39 (8.8)	236 (53.5)	3.10	0.8000 (0.6891 – 0.9109)	0.0001 (Sig)
Alimi et al^5^ 2013 (Neuro-Iran)	1178^‡^	74 (6.3)	71 (6.0)	57 (4.8)	279 (23.7)	3.12	0.7800 (0.6597 – 0.9003)	0.0001(Sig)

* Including neurosurgery journals,

† Excluding neurosurgery topics,

‡ Only 481 articles were scored.

IF - impact factor, Sig - Significant, NS - not significant, Ortho - orthopedics, Neuro - Neurosurgery

As a result of the exclusion of letters to editors and bench research there were no articles with LOE V in our study, which is comparable to the other Saudi reports.^1^,^2^ Case reports accounted for 47.5% of articles, which is higher than the 13-41.9% quoted by others.^1^-^5^ Commonly case reports are not included in the LOE grading because many believe that they are weak in assessing the efficacy of treatment.^3^,^4^ We included them because they represented a substantial portion of the total KSA neurosurgery research output. We observed a significantly higher LOE in articles that were published during 2001-2013 compared with those that were published from 1990-2000. Such a finding, not observed in the Saudi literature,^1^,^2^ is encouraging and may reflect increasing awareness of EBM by Saudi neurosurgeons. Two hospitals in Riyadh (KKUH and KFSHRC) accounted for 61% of the total publications. This reflects an imbalance between various KSA neurosurgical centers in their research contribution, which may be related to differences in experience, facilities, and clinical load. Alimi et al^5^ reported a larger contribution to research by Iranian neurosurgeons working in universities and in major cities. Collaboration with international centers had a positive influence on the LOE and should be encouraged.

Neurosciences (Riyadh) was the most frequently used journal, and most of the articles were published in non-neurosurgical journals. Only 26% were published in Saudi Journals, which is lower than what others have cited for publications in local journals.^1^,^5^ The articles were published in 99 journals and despite most of these journals having acceptable IFs, their large number is a signal that the KSA neurosurgical research needs to be more focused. We found that neither the journal’s category nor its IF affected the LOE. Amiri et al^3^ reported a significant variation in the LOE between articles published in 5 major spine journals that were consistent with their IFs. Rothoerl et al^4^ on the other hand, found comparable LOE among articles published in 3 major neurosurgical journals that had different IFs. The most common research topics in KSA clinical neurosurgery publications were tumor and pediatric neurosurgery. Infection was well represented at 10.1%, and trauma at 7.7% was under-represented compared with others.^5^ Neurosciences topics (non-neurosurgical) accounted for 16.2% of publications and were associated with a significantly higher LOE. This would indicate a positive aspect to research collaboration between neurosurgeons and their neurosciences colleagues. The median citation numbers of the articles was 11, and we found no significant difference in the LOE between articles that had citation numbers equal and higher or lower than 10. It is recognized that at times articles reporting case series and retrospective studies are the only source of data available so they become well cited.^3^

Comparison of our findings with the LOE in publications from other KSA surgical specialties (**Table 1**) showed that the neurosurgical LOE results were comparable to plastic surgery,^2^ but significantly lower than orthopedic surgery,^1^ despite the LOE in the latter 2 reported as comparable.^2^ In addition, our LOE was significantly lower when compared with publications in the international neurosurgical literature,^4^ in the international spine literature,^3^ and in publications by Iranian neurosurgeons.^5^ The LOE of clinical neurosurgery research in KSA over 24 years was low (level IV 91%, and prospective studies 6.4%). It is accepted that not all clinical research can be constructed in the method required for it to be classified as high LOE.^1^-^3^ Nevertheless, it would be reasonable to assume that the majority of KSA neurosurgeons find the execution of high LOE clinical research difficult. The reasons are multifactorial, and include lack of experience, time, interest as well as limited logistic and financial support. In addition, the absence of qualified Saudi neurosurgeons holding research degrees as well as the dilution of the clinical load due to the non-regionalization of the service can be considered as barriers to conducting quality neurosurgical research in KSA.

There are limitations in the interpretation of our results. Some publications may have been missed due to search errors as a result of the misspelling of names and because the publications were in journals that are not indexed in “PubMed” and “Google Scholar”. We believe this was minimized by searching in 2 databases. Relevant studies were unlikely to be missed as most articles had more than one Saudi author.

In conclusion, this study demonstrates a relatively low LOE of clinical neurosurgery in KSA compared with the literature. The LOE was significantly higher over the last decade, in the presence of international collaboration, and when the research topic was related to other neurosciences. The practice of EBM should be encouraged and local journals should consider the LOE of articles during the review process. More academic units should be established in KSA, and neurosurgeons should aim at producing more high quality neurosurgical research and less case reports.
